# Supplementary material for: Origin and Evolution of the Human Bcl2-Associated Athanogene-1 (BAG-1)
Source: Int J Mol Sci. 2020 Dec 18;21(24):9701. doi: 10.3390/ijms21249701 (PMC7766421; doi:10.3390/ijms21249701)
Supplement: Supplementary file 1 [file ijms-21-09701-s001.zip › Supplementary_Information_R1.pdf]

Origin and Evolution of the Human Bcl2 Associated Athanogene -1 (BAG-1)

Peter Nguyen 1‡, Kyle Hess 2‡, Larissa Smulders1, Dat Le1, Carolina Briseno1, Christina M. Chavez 1, and Nikolas Nikolaidis 1,*
1 Department of Biological Science, Center for Applied Biotechnology Studies, and Center for Computational and Applied Mathematics, College of Natural Sciences and Mathematics, California State University Fullerton, Fullerton, CA 92834-6850, USA; peter_nguyen@Fullerton.edu (P.N); l.smulders@csu.fullerton.edu (L.S.); datqle@yahoo.com (D.L); cbriseno89@csu.fullerton.edu (C.B.); christinachavez@csu.fullerton.edu (C.M.C.)
2 Department of Genome Sciences, Molecular and Cellular Biology Graduate Program, University of Washington, Seattle, WA; kylehess@uw.edu (K.H.)
‡ These authors contributed equally to this work
* Correspondence: nnikolaidis@fullerton.edu; Tel.: +1-657-278-4526 (N.N.)

Supplementary Information

Supplementary Table S1

Supplementary Table S2

Supplementary Figure S1

Supplementary Figure S2

Supplementary Figure S3

Supplementary Table S1. This table shows the accession numbers of the sequences as well as the scientific and common names of the species used in the analyses presented in Figure 3

Species	Name	Accession Number
Homo sapiens	human	NP_001165886.1
Callithrix jacchus	marmoset	XP_002743090.1
Canis lupus familiaris	dog	XP_005627044.1
Mus musculus	mouse	NP_033866.4
Amazona aestiva	blue-fronted parrot	KQK82629.1
Gallus gallus	chicken	NP_001103162.1
Chelonia mydas	Green sea turtle	XP_007058982.1
Callorhinchus milii	Australian ghost shark	AFP09125.1
Astyanax mexicanus	Mexican tetra-Cave fish	XP_007260231.1
Danio rerio	zebrafish	NP_001092206.1
Priapulus caudatus	penis worm	XP_014668103.1
Hydra vulgaris	fresh-water polyp	XP_002160797.2
Strongylocentrotus purpuratus	Pacific purple sea urchin	XP_784685.2
Crassostrea gigas	Pacific oyster	EKC35341.1
Daphnia pulex	water flea	EFX77677.1
Caenorhabditis briggsae	nematode	XP_002640155.1
Caenorhabditis elegans	nematode	NP_491893.1
Arabidopsis thaliana	thale cress	NP_200019.2
Brassica rapa	field mustard	XP_009132555.1
Morus notabilis	mulberry tree	XP_010086544.1
Zea mays	corn	NP_001141543.1
Fragaria vesca	strawberry	NP_001292200.1
Schizosaccharomyces pombe 972h-	fission yeast	NP_596760.1
Schizosaccharomyces octosporus yFS286	fission yeast	XP_013018865.1
Schizosaccharomyces cryophilus OY26	fission yeast	XP_013024760.1

Supplementary Table S2. Statistical analyses (Tukey HSD) of the experiments shown in Figures 13 and 14

**P-values of Figure 13a**			
		**Tukey HSD**	**Tukey HSD**
	**Pair**	**p-value**	**inference**
0-30	A vs B	0.0021052	** p<0.01
	A vs C	0.0010053	** p<0.01
	A vs D	0.0010053	** p<0.01
	A vs E	0.0010053	** p<0.01
	A vs F	0.1354966	insignificant
	A vs G	0.0010053	** p<0.01
0-60	A vs B	0.0010053	** p<0.01
	A vs C	0.0010053	** p<0.01
	A vs D	0.0010053	** p<0.01
	A vs E	0.0010053	** p<0.01
	A vs F	0.0010053	** p<0.01
	A vs G	0.0010053	** p<0.01
0-90	A vs B	0.8760277	insignificant
	A vs C	0.0010053	** p<0.01
	A vs D	0.038334	* p<0.05
	A vs E	0.0010053	** p<0.01
	A vs F	0.1076909	insignificant
	A vs G	0.0010053	** p<0.01
**P-values of Figure 13b**			
0-30	A vs B	0.0010053	** p<0.01
	A vs C	0.6743635	insignificant
	A vs D	0.0010053	** p<0.01
	A vs E	0.8999947	insignificant
	A vs F	0.6276	insignificant
	A vs G	0.0010053	** p<0.01
0-60	A vs B	0.0010053	** p<0.01
	A vs C	0.2832351	insignificant
	A vs D	0.0283989	* p<0.05
	A vs E	0.8999947	insignificant
	A vs F	0.1853428	insignificant
	A vs G	0.4501726	insignificant
0-90	A vs B	0.0010053	** p<0.01
	A vs C	0.7385733	insignificant
	A vs D	0.0010053	** p<0.01
	A vs E	0.0123581	* p<0.05

	A vs F	0.0022071	** p<0.01
	A vs G	0.8999947	insignificant

Legend:	
A1A only	A
A1A + BAGS WT	B
A1A + BAGS 215	C
A1A + BAGS 216	D
A1A + BAGS 219	E
A1A + BAGS 229	F
A1A + BAGS 233	G

P-values of Figure 14

	Pair	Tukey HSD p-value	Tukey HSD inference
	A vs B	0.0010053	** p<0.01
	A vs C	0.8999947	insignificant
	A vs D	0.5651691	insignificant
	A vs E	0.0010053	** p<0.01
	A vs F	0.3643165	insignificant
	A vs G	0.4989294	insignificant
	A vs H	0.380636	insignificant
	A vs I	0.431198	insignificant
	C vs D	0.2517504	insignificant
	C vs E	0.0010053	** p<0.01
	C vs F	0.134966	insignificant
	C vs G	0.206425	insignificant
	C vs H	0.1426405	insignificant
	C vs I	0.1678717	insignificant

Legend:	
GFP	A
A1A	B
BAG	C
A1A+BAG	D
A1A+215	E
A1A+216	F

A1A+219	G
A1A+229	H
A1A+233	I

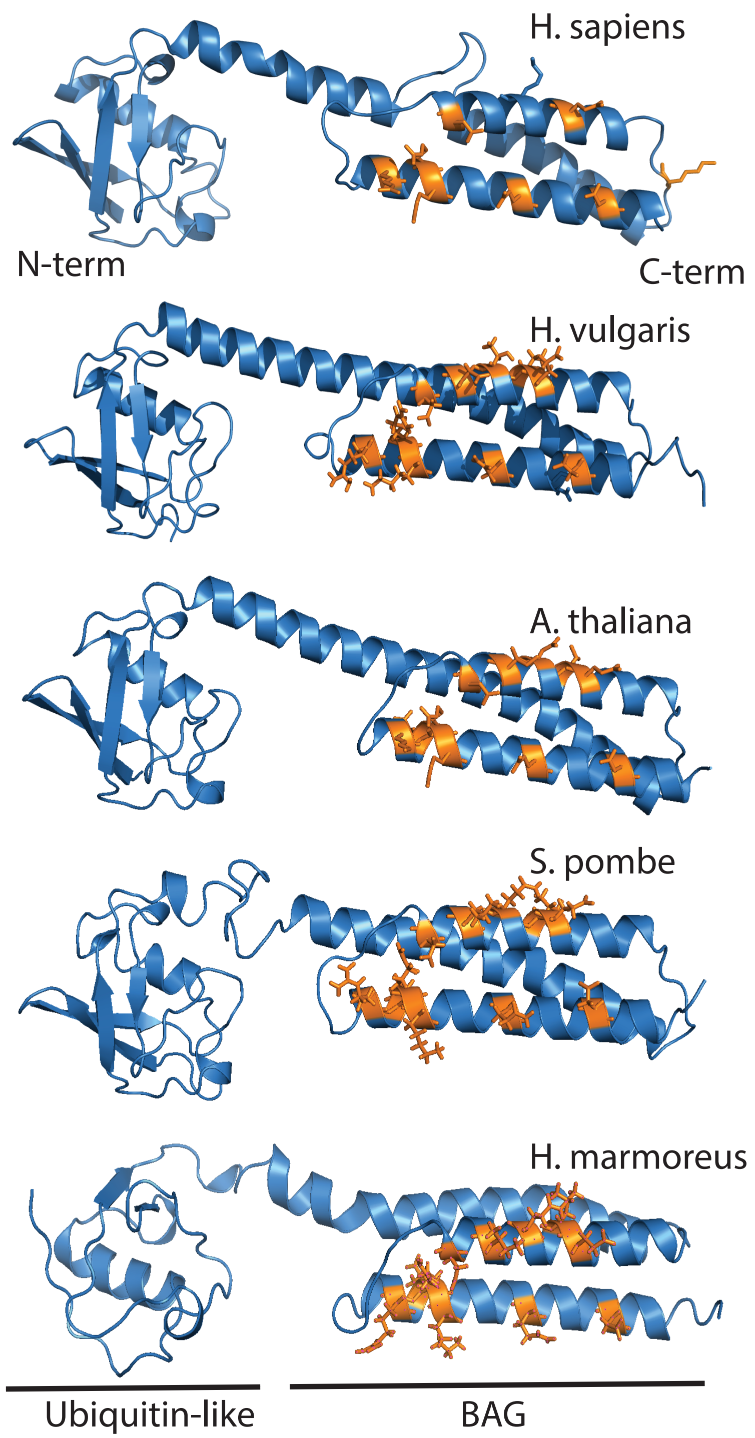

Supplementary Figure S1. Divergent Bag-1 homologs are structurally-conserved and maintain positioning of key Hsp70 interaction sites. Bag-1 protein structure for four representative species was generated using SWISS-Model. Protein structures were rendered and analyzed using PyMOL 1.3. Amino acid residues highlighted in orange represent Hsp70 interaction sites.

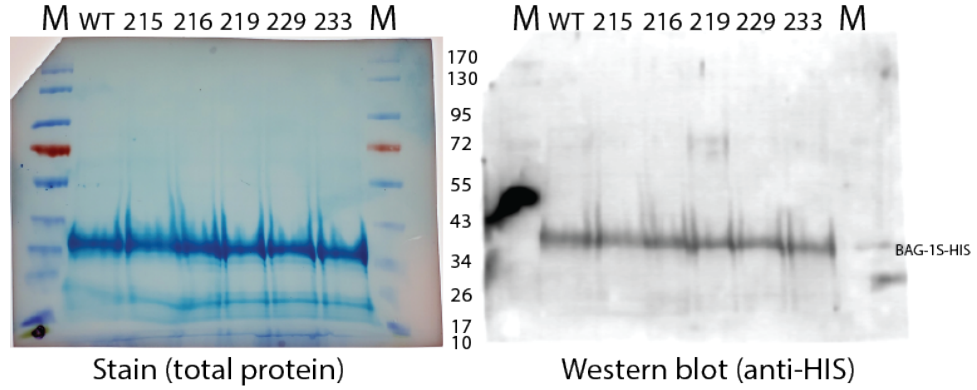

Supplementary Figure S2. Purified recombinant proteins corresponding to the wild type (WT) and mutated BAG-1 recombinant proteins. Approximately 8μg protein was loaded on an SDS-PAGE gel. After the proteins were transferred to a nitrocellulose membrane, the membrane was stained with coomassie blue stain (left image); followed by Western blotting using an antibody against the poly-Histidine tag (right image). The blot was stained for total protein with the Pierce™ Reversible Protein Stain (Thermo Scientific™; Waltham, MA) and the western using an antibody from Cell Signaling (Danvers, MA; antibody #2365; at a dilution of 1:1000). The western signals were detected using either the Omega Lum C (Gel Company; San Francisco, CA) or the Ci-Digit (LicoR; Lincoln, NE) systems.

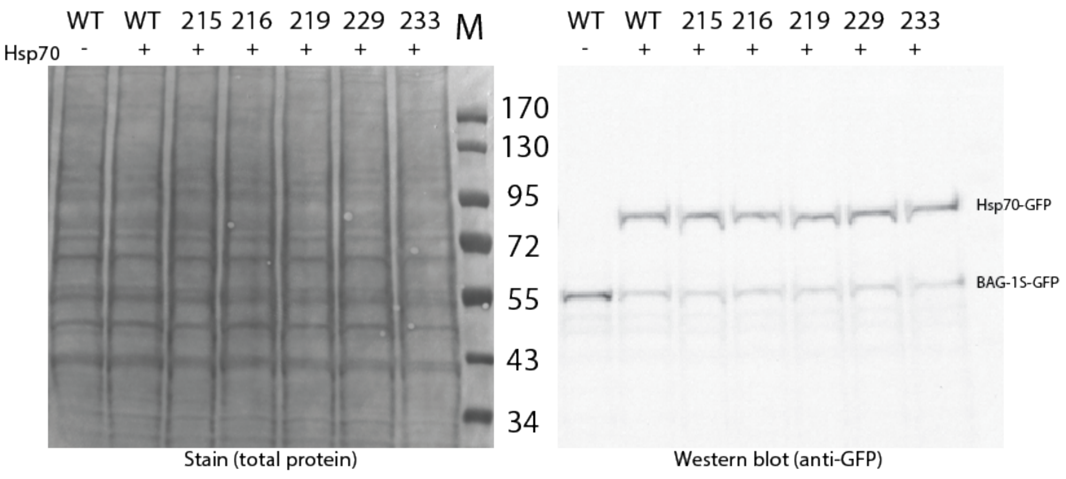

Supplementary Figure S3. Western blotting analysis of the cell samples used in the refolding assay pictured in Figure 14. The cells were transfected with 1 μg/ml total DNA. In particular, 0.5 μg/ml of firefly luciferase and 0.5 μg/ml WT-BAG-1-GFP; 0.33 μg/ml of firefly luciferase, 0.33 μg/ml HSPA1A-GFP, and 0.33 μg/ml of the WT or mutant BAG-1-GFP. The blots were stained for total protein with the Pierce™ Reversible Protein Stain (Thermo Scientific™; Waltham, MA). The western signals were detected using either the Omega Lum C (Gel Company; San Francisco, CA) or the Ci-Digit (LicoR; Lincoln, NE) systems.
