# Supplementary material for: Origin and Evolution of the Human Bcl2-Associated Athanogene-1 (BAG-1)
Source: Int J Mol Sci. 2020 Dec 18;21(24):9701. doi: 10.3390/ijms21249701 (PMC7766421; doi:10.3390/ijms21249701)
Supplement: Supplementary file 1 [file ijms-21-09701-s001.zip › Supplementary_Information_R1_track.docx]

Origin and Evolution of the Human Bcl2 Associated Athanogene -1 (BAG-1)

Peter Nguyen 1‡, Kyle Hess 2‡, Larissa Smulders1, Dat Le1, Carolina Briseno1, Christina M. Chavez 1, and Nikolas Nikolaidis 1,*

1 Department of Biological Science, Center for Applied Biotechnology Studies, and Center for Computational and Applied Mathematics, College of Natural Sciences and Mathematics, California State University Fullerton, Fullerton, CA 92834-6850, USA; peter_nguyen@Fullerton.edu (P.N); l.smulders@csu.fullerton.edu (L.S.); datqle@yahoo.com (D.L); cbriseno89@csu.fullerton.edu (C.B.); christinachavez@csu.fullerton.edu (C.M.C.)

2 Department of Genome Sciences, Molecular and Cellular Biology Graduate Program, University of Washington, Seattle, WA; kylehess@uw.edu (K.H.)

‡ These authors contributed equally to this work

* Correspondence: nnikolaidis@fullerton.edu; Tel.: +1-657-278-4526 (N.N.)

**Supplementary Information**

Supplementary Table S1

Supplementary Table S2

Supplementary Figure S1

Supplementary Figure S2

Supplementary Figure S3

**Supplementary Table S1.** This table shows the accession numbers of the sequences as well as the scientific and common names of the species used in the analyses presented in Figure 3

| **Species** | **Name** | **Accession Number** |
| --- | --- | --- |
| *Homo sapiens* | human | NP_001165886.1 |
| *Callithrix jacchus* | marmoset | XP_002743090.1 |
| *Canis lupus familiaris* | dog | XP_005627044.1 |
| *Mus musculus* | mouse | NP_033866.4 |
| *Amazona aestiva* | blue-fronted parrot | KQK82629.1 |
| *Gallus gallus* | chicken | NP_001103162.1 |
| *Chelonia mydas* | Green sea turtle | XP_007058982.1 |
| *Callorhinchus milii* | Australian ghost shark | AFP09125.1 |
| *Astyanax mexicanus* | Mexican tetra-Cave fish | XP_007260231.1 |
| *Danio rerio* | zebrafish | NP_001092206.1 |
| *Priapulus caudatus* | penis worm | XP_014668103.1 |
| *Hydra vulgaris* | fresh-water polyp | XP_002160797.2 |
| *Strongylocentrotus purpuratus* | Pacific purple sea urchin | XP_784685.2 |
| *Crassostrea gigas* | Pacific oyster | EKC35341.1 |
| *Daphnia pulex* | water flea | EFX77677.1 |
| *Caenorhabditis briggsae* | nematode | XP_002640155.1 |
| *Caenorhabditis elegans* | nematode | NP_491893.1 |
| *Arabidopsis thaliana* | thale cress | NP_200019.2 |
| *Brassica rapa* | field mustard | XP_009132555.1 |
| *Morus notabilis* | mulberry tree | XP_010086544.1 |
| *Zea mays* | corn | NP_001141543.1 |
| *Fragaria vesca* | strawberry | NP_001292200.1 |
| *Schizosaccharomyces pombe 972h-* | fission yeast | NP_596760.1 |
| *Schizosaccharomyces octosporus yFS286* | fission yeast | XP_013018865.1 |
| *Schizosaccharomyces cryophilus OY26* | fission yeast | XP_013024760.1 |

**Supplementary Table S2.** Statistical analyses (Tukey HSD) of the experiments shown in Figures 13 and 14

| **P-values of Figure 13a** | |  |  |
| --- | --- | --- | --- |
|  |  | **Tukey HSD** | **Tukey HSD** |
|  | **Pair** | **p-value** | **inference** |
| 0-30 | A vs B | 0.0021052 | ** p<0.01 |
|  | A vs C | 0.0010053 | ** p<0.01 |
|  | A vs D | 0.0010053 | ** p<0.01 |
|  | A vs E | 0.0010053 | ** p<0.01 |
|  | A vs F | 0.1354966 | insignificant |
|  | A vs G | 0.0010053 | ** p<0.01 |
| 0-60 | A vs B | 0.0010053 | ** p<0.01 |
|  | A vs C | 0.0010053 | ** p<0.01 |
|  | A vs D | 0.0010053 | ** p<0.01 |
|  | A vs E | 0.0010053 | ** p<0.01 |
|  | A vs F | 0.0010053 | ** p<0.01 |
|  | A vs G | 0.0010053 | ** p<0.01 |
| 0-90 | A vs B | 0.8760277 | insignificant |
|  | A vs C | 0.0010053 | ** p<0.01 |
|  | A vs D | 0.038334 | * p<0.05 |
|  | A vs E | 0.0010053 | ** p<0.01 |
|  | A vs F | 0.1076909 | insignificant |
|  | A vs G | 0.0010053 | ** p<0.01 |
| **P-values of Figure 13b** | |  |  |
| 0-30 | A vs B | 0.0010053 | ** p<0.01 |
|  | A vs C | 0.6743635 | insignificant |
|  | A vs D | 0.0010053 | ** p<0.01 |
|  | A vs E | 0.8999947 | insignificant |
|  | A vs F | 0.6276 | insignificant |
|  | A vs G | 0.0010053 | ** p<0.01 |
| 0-60 | A vs B | 0.0010053 | ** p<0.01 |
|  | A vs C | 0.2832351 | insignificant |
|  | A vs D | 0.0283989 | * p<0.05 |
|  | A vs E | 0.8999947 | insignificant |
|  | A vs F | 0.1853428 | insignificant |
|  | A vs G | 0.4501726 | insignificant |
| 0-90 | A vs B | 0.0010053 | ** p<0.01 |
|  | A vs C | 0.7385733 | insignificant |
|  | A vs D | 0.0010053 | ** p<0.01 |
|  | A vs E | 0.0123581 | * p<0.05 |
|  | A vs F | 0.0022071 | ** p<0.01 |
|  | A vs G | 0.8999947 | insignificant |

| Legend: |  |
| --- | --- |
|  |  |
| A1A only | A |
| A1A + BAGS WT | B |
| A1A + BAGS 215 | C |
| A1A + BAGS 216 | D |
| A1A + BAGS 219 | E |
| A1A + BAGS 229 | F |
| A1A + BAGS 233 | G |

| **P-values of Figure 14** | |  |  |  |
| --- | --- | --- | --- | --- |
|  |  |  | **Tukey HSD** | **Tukey HSD** |
|  |  | **Pair** | **p-value** | **inference** |
|  |  | A vs B | 0.0010053 | ** p<0.01 |
|  |  | A vs C | 0.8999947 | insignificant |
|  |  | A vs D | 0.5651691 | insignificant |
|  |  | A vs E | 0.0010053 | ** p<0.01 |
|  |  | A vs F | 0.3643165 | insignificant |
|  |  | A vs G | 0.4989294 | insignificant |
|  |  | A vs H | 0.380636 | insignificant |
|  |  | A vs I | 0.431198 | insignificant |
|  |  | C vs D | 0.2517504 | insignificant |
|  |  | C vs E | 0.0010053 | ** p<0.01 |
|  |  | C vs F | 0.134966 | insignificant |
|  |  | C vs G | 0.206425 | insignificant |
|  |  | C vs H | 0.1426405 | insignificant |
|  |  | C vs I | 0.1678717 | insignificant |

| Legend: |  |
| --- | --- |
|  |  |
| GFP | A |
| A1A | B |
| BAG | C |
| A1A+BAG | D |
| A1A+215 | E |
| A1A+216 | F |
| A1A+219 | G |
| A1A+229 | H |
| A1A+233 | I |

**Supplementary Figure S1.** Divergent Bag-1 homologs are structurally-conserved and maintain positioning of key Hsp70 interaction sites. Bag-1 protein structure for four representative species was generated using SWISS-Model. Protein structures were rendered and analyzed using PyMOL 1.3. Amino acid residues highlighted in orange represent Hsp70 interaction sites.

**
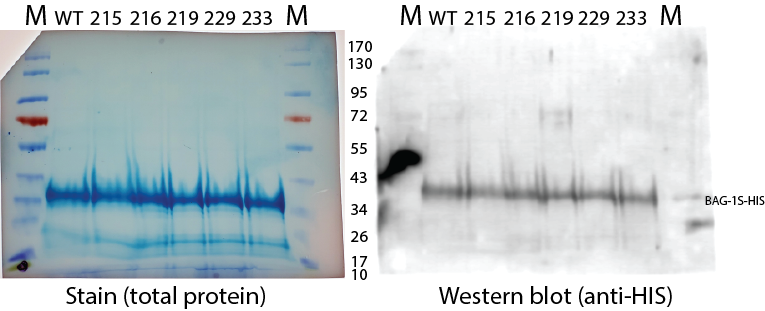
**

**Supplementary Figure S2.** Purified recombinant proteins corresponding to the wild type (WT) and mutated BAG-1 recombinant proteins. Approximately 8μg protein was loaded on an SDS-PAGE gel. After the proteins were transferred to a nitrocellulose membrane, the membrane was stained with coomassie blue stain (left image); followed by Western blotting using an antibody against the poly-Histidine tag (right image). The blot was stained for total protein with the Pierce™ Reversible Protein Stain (Thermo Scientific™; Waltham, MA) and the western using an antibody from Cell Signaling (Danvers, MA; antibody #2365; at a dilution of 1:1000). The western signals were detected using either the Omega Lum C (Gel Company; San Francisco, CA) or the Ci-Digit (LicoR; Lincoln, NE) systems.

**
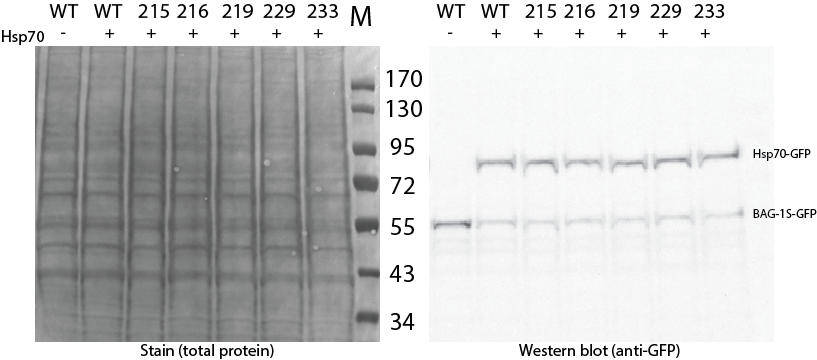
**

**Supplementary Figure S3.** Western blotting analysis of the cell samples used in the refolding assay pictured in Figure 14. The cells were transfected with 1 µg/ml total DNA. In particular, 0.5 µg/ml of firefly luciferase and 0.5 µg/ml WT-BAG-1-GFP; 0.33 µg/ml of firefly luciferase, 0.33 µg/ml HSPA1A-GFP, and 0.33 µg/ml of the WT or mutant BAG-1-GFP. The blots were stained for total protein with the Pierce™ Reversible Protein Stain (Thermo Scientific™; Waltham, MA). The western signals were detected using either the Omega Lum C (Gel Company; San Francisco, CA) or the Ci-Digit (LicoR; Lincoln, NE) systems.
